# Prognosis Implication of a Novel Metabolism-Related Gene Signature in Ewing Sarcoma

**DOI:** 10.1155/2021/3578949

**Published:** 2021-12-10

**Authors:** Zhuo-Yuan Chen, Huiqin Yang, Jie Bu, Qiong Chen, Zhen Yang, Hui Li

**Affiliations:** ^1^Department of Orthopedics, The Second Xiangya Hospital, Central South University, 139th Renmin Middle Road, Changsha, Hunan, China; ^2^Orthopedic Biomedical Materials Engineering Laboratory of Hunan Province, 139th Renmin Middle Road, Changsha, Hunan, China; ^3^Hunan Cancer Hospital and the Affiliated Cancer Hospital of Xiangya School of Medicine, Central South University, 283 Tongzipo Road, Changsha, Hunan, China; ^4^Department of Ultrasound, Wuhan No. 1 Hospital, 215 Zhongshan Dadao, Wuhan, Hubei, China; ^5^Department of Radiology, Hubei Cancer Hospital, 116 Zhuodaoquan South Road, Wuhan, Hubei, China

## Abstract

Ewing sarcoma (ES) is one of the most common bone cancers in adolescents and children. Growing evidence supports the view that metabolism pathways play critical roles in numerous cancers (He et al. (2020)). However, the correlation between metabolism-associated genes (MTGs) and Ewing sarcoma has not been investigated systematically. Here, based on the univariate Cox regression analysis, we get survival genes from differentially expressed genes (DEGs) from Gene Expression Omnibus (GEO) cohort. Multivariate Cox regression analysis and least absolute shrinkage and selection operator (LASSO) regression analysis were employed to establish the MTG signature. Comprehensive survival analyses including receiver operating characteristic (ROC) curves and Kaplan–Meier analysis were applied to estimate the independent prognostic value of the signature. The ICGC cohort served as the validation cohort. A nomogram was constructed based on the risk score of the MTG signature and other independent clinical variables. The CIBERSORT algorithm was applied to estimate immune infiltration. In addition, we explored the correlation between MTG signature and immune checkpoints. Collectively, this work presents a novel MTG signature for prognostic prediction of Ewing sarcoma. It also suggests six genes that are potential prognostic indicators and therapeutic targets for ES.

## 1. Introduction

Ewing sarcoma is a primary malignancy of the bone or soft tissue [1]. It is ranked the second most prevalent bone cancer in adolescents and children [2]. Current evidence shows that metastasis of ES is still the main indicator to predict the outcomes of ES patients for lacking effective biomarkers [3]. Koustas et al. found that the 5-year survival rate of patients with metastasis is only 20%–45% [4]. Therefore, effective biomarkers that can accurately predict disease outcomes and offer novel therapeutic targets for Ewing sarcoma are urgently needed.

The concept of metabolic reprogramming was first put forward by Otto Warburg in 1924 [5]. Several lines of evidence have demonstrated metabolism as among the most compelling traits in cancers, for it is associated with various biological processes, including growth, proliferation, migration, and invasion, and angiogenesis [6, 7]. Cancer cells can adjust their metabolic patterns to guarantee sufficient energy and substance. A previous study revealed that the restoration or blockage of metabolic pathways may be a promising therapeutic strategy for tumors [8]. In the context of Ewing sarcoma, a number of studies explored the importance of metabolic reprogramming in disease progression and prognosis. Tanner et al. [9] found that EWS/FLI could induce diversion of metabolites toward oncogenesis-related biosynthetic pathways to meet the demand of ES cells, for example, by shunting glycolytic intermediates into serine and glycine synthesis. The inhibitors of the glycolytic enzyme lactate dehydrogenase (LDH), which are critical in cellular metabolism, are potential therapeutic targets for ES treatment [10]. Hence, uncovering metabolism-related biomarkers greatly sheds light on ES diagnosis and prognosis.

In this study, we compared the normal and ES tissues to reveal the DEGs of MTGs. Based on DEGs and prognosis-related genes, a metabolic gene signature was established, which allowed us to stratify patients into high- and low-risk subtypes and ensure accurate prediction of survival prognosis. Furthermore, the model was validated with an independent cohort from the International Cancer Genome Consortium (ICGC) database. Analysis of the correlation between the subtypes with immune infiltration was achieved through single-sample gene set enrichment analysis (ssGSEA) to explore the roles of 24 immune cells in the metabolism-related signature. In summary, we have constructed a prognostic model that can accurately predict ES prognosis.

## 2. Materials and Methods

### 2.1. Data Collection

We downloaded 944 MTGs from the Kyoto Encyclopedia of Genes and Genomes (KEGG) pathways as previously described by Chao-Yang et al. [11]. The transcriptome profiles and related clinical data of ES patients were extracted from the GEO database. In total, 18 normal samples (skeletal muscle) and 64 Ewing sarcoma samples from the dataset (GSE17679) were analyzed. Fifty-five samples contained both transcriptome and clinical data in the validation ICGC cohort.

### 2.2. Construction and Validation of the Metabolism-Related Gene Signature

The “limma” R package was employed to obtain DEGs (FDR <0.05) from the GEO dataset while genes related to overall survival (OS) were identified with univariate Cox regression analysis. By taking the intersection of DEGs and prognosis-related genes, 255 genes were identified for further analysis. According to multivariate Cox regression analysis, 6 genes were retrieved. Next, the least absolute shrinkage and selection operator (LASSO) regression analysis was employed to construct the prognostic model of the multivariate Cox regression results. The risk score was calculated using the following formula: risk score = ∑icoefficient (genei) × expression (genei). ES patients were classified into high-risk and low-risk groups based on the median risk score. Furthermore, “survival,” “survivalROC,” and “stats” packages were applied to draw the Kaplan–Meier survival curves and conduct time-dependent ROC curve analysis and principal component analysis (PCA), respectively. Multivariate and univariate Cox regression analyses were applied to examine the effectiveness of the risk score as an independent prognostic indicator using the risk score and available clinicopathological data. Based on the risk score, ES patients from the ICGC dataset were classified into high- and low-risk groups and analyses were conducted to validate the effectiveness.

### 2.3. Gene Set Enrichment Analysis

Based on the packages “GSVA” and “GSEABase,” the Gene Set Enrichment Analysis (GSEA) was applied to explore the biological functions and pathways according to the low- and high-risk groups (*p* value was set as 0.05).

### 2.4. Evaluation of the Prognostic Signature and the Construction of Nomogram

The predictive ability of the prognostic model for different clinicopathological characteristics was explored in the GEO cohort. Based on the “rms” R package, the nomogram was performed by the overall survival data of the GEO dataset. Moreover, the 3- and 5-year calibration plots were applied to assess the accuracy of the nomogram.

### 2.5. Evaluation of Immune Cell Infiltration and Immune Checkpoints

We analyzed the correlation between the model and immune cell infiltration using the CIBERSORT algorithm [12] to identify the 22 immune cells' fractions in the GEO dataset. The relationship between 22 immune cells was evaluated with the Pearson correlation analysis. Furthermore, we obtained the differential immune cells by comparing the high- and low-risk groups. Later, Kaplan–Meier analysis was employed to analyze the correlation between the differential immune cells and ES patient prognosis. In addition, we estimated the association between MTG signature and immune checkpoints via the expression levels of immune checkpoint genes.

### 2.6. Statistical Analysis

Student's *t*-test was applied to identify the DEGs in the GEO cohort. Wilcoxon test and chi-square test were applied to analyze continuous and categorical variables, respectively. K-M curve and the log-rank test were performed to evaluate the differences in OS. All statistical analyses were performed with the R software (version 4.0.1), and *p* < 0.05 denoted statistical significance.

## 3. Results

### 3.1. Identification of Metabolism-Related Prognostic DEGs and Construction of the Signature

**Figure 1:**
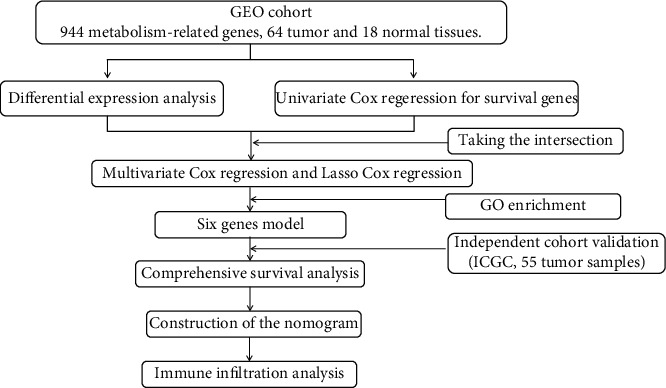
Flowchart of data collection and analysis.

A schematic representation of the study is illustrated in Figure 1. The characteristics of ES patients in the two datasets are listed in Table 1. The retrieved MTGs are displayed in Supplementary Table 1.

**Figure 2:**
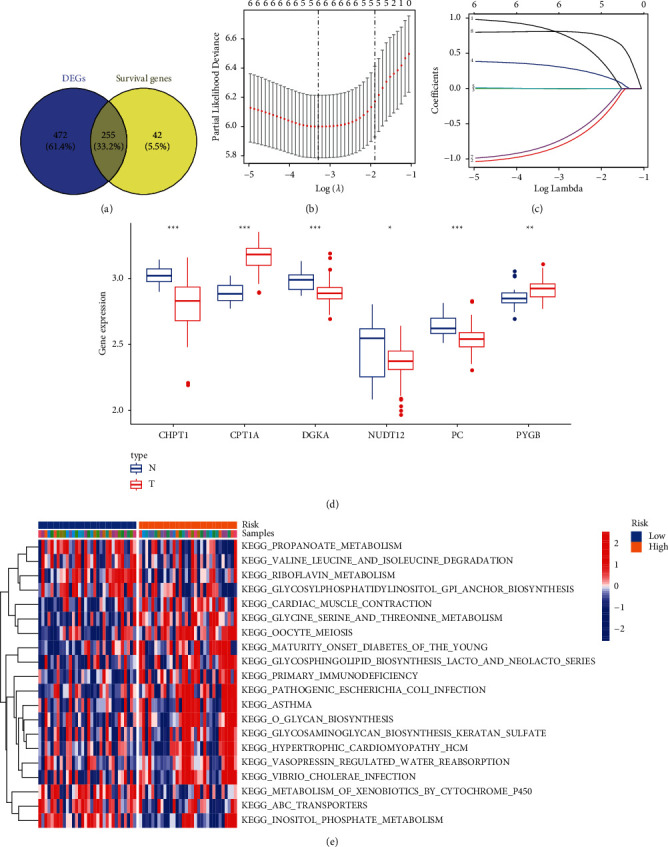
Identification of the candidate MTGs in the GEO dataset. (a) Venn diagram showing the intersection between DEGs and the survival-related genes. (b, c) LASSO and Cox regression analyses. (d) Boxplot to display the differential expression of genes in MTG signature. (e) The GSEA for the GEO cohort.

Of note, 727 MTGs were differentially expressed between the ES tissues and the normal tissues. Based on the univariate Cox regression analysis results, we obtained 297 prognostic MTGs which were related to OS. Additionally, 255 intersection results (Figure 2(a)) of DEGs and prognostic genes were illustrated in a Venn diagram. Based on the multivariate Cox regression analysis results, 255 genes were screened, 6 of which were highly related to OS. Through the LASSO regression analysis (Figures 2(b) and 2(c)), we constructed the MTG prognostic signature and applied it to calculate the risk score using the following formula: risk score = (0.7457^*∗*^ expression of PC) + (−0.7713^*∗*^ expression of DGKA) + (−0.0036^*∗*^ expression of CPT1A) + (0.2923^*∗*^ expression of CHPT1) + (−0.6910^*∗*^ expression of NUDT12) + (0.8069^*∗*^ expression of PYGB). Subsequently, ES patients were classified into the low-risk group (*n* = 32) and the high-risk group (*n* = 32) according to the median cutoff risk score. As depicted in the box plot (Figure 2(d)), all the six genes were differentially expressed between the ES tissues and the normal tissues. GSEA outcomes (Figure 2(e)) indicated that the top 5 were glycine, serine, and threonine metabolism, oocyte meiosis, maturity-onset diabetes of the young, cardiac muscle contraction, and vasopressin-regulated water reabsorption.

### 3.2. Verification of the MTG Signature

**Figure 3:**
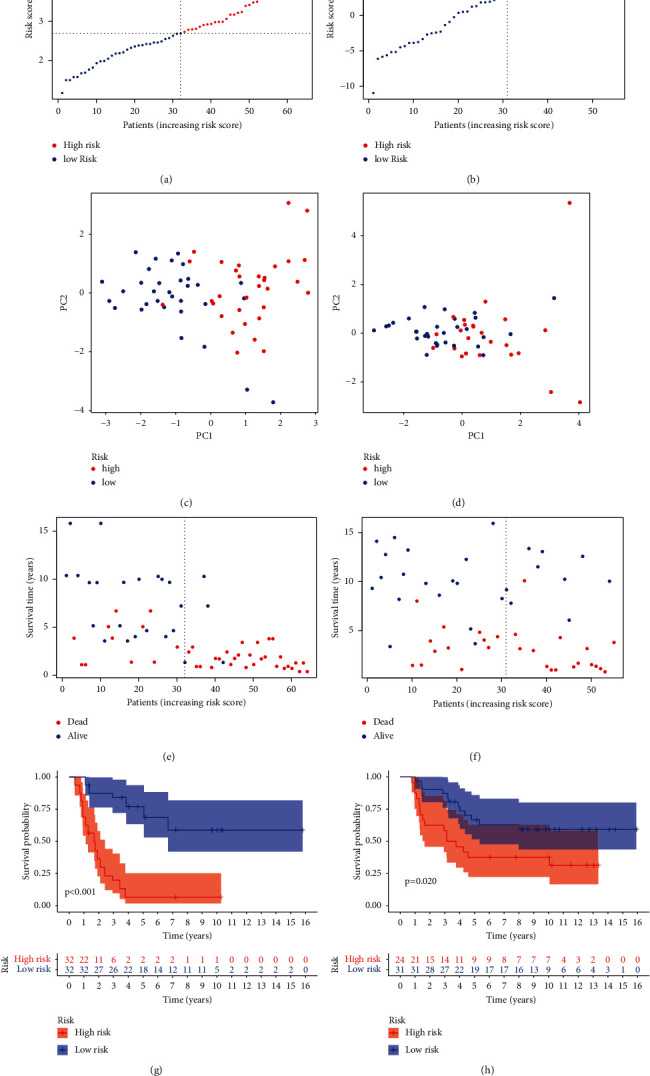
Characteristics of the risk score of the MTG signature. The distribution of the risk scores, survival status, PCA analysis, and K-M survival analysis in GEO (a, c, e, g) and ICGC (b, d, f, h) cohorts.

Thirty-two high-risk patients and 25 low-risk patients were found in the GEO dataset, while 30 high-risk patients and 25 low-risk patients were found in the ICGC dataset (Figures 3(a) and 3(b)). The outcomes of PCA verified that the two groups were mainly distributed in two different directions (Figures 3(c) and 3(d)). The status of ES patients in the GEO and ICGC datasets is described in Figures 3(e) and 3(f). These data demonstrate that the high-risk groups were correlated with more deaths. Besides, Kaplan–Meier curve analysis (Figures 3(g) and 3(h)) was applied to demonstrate the OS difference between the two risk groups. The *P* value of GEO and ICGC datasets was statistically significant (<0.001 and 0.020).

### 3.3. Strong Prognostic Power of the MTG Signature

**Figure 4:**
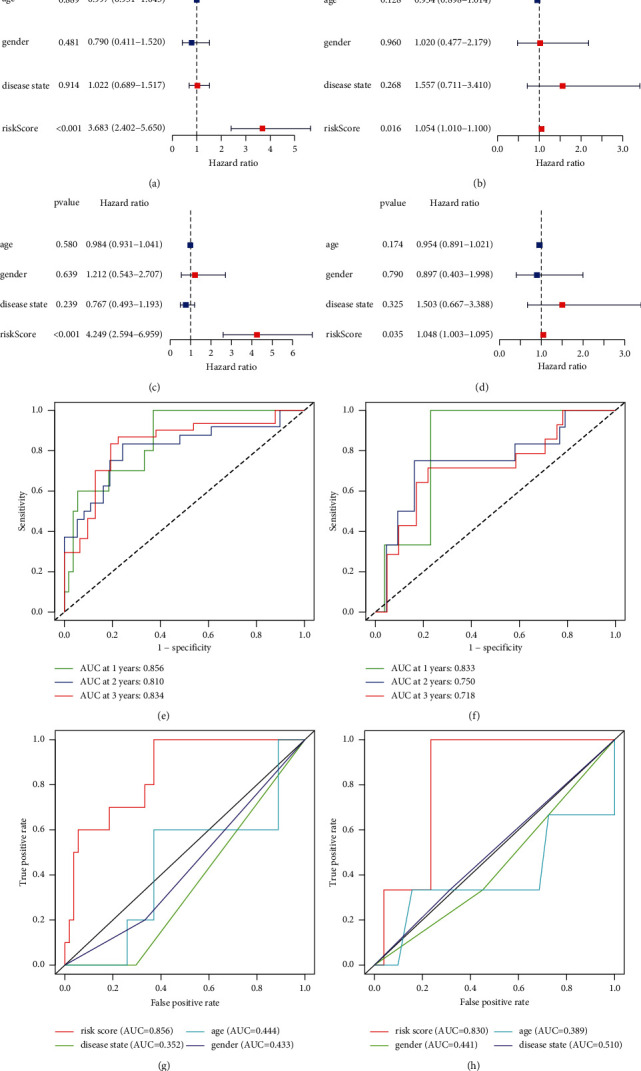
Comprehensive survival analysis of the MTG signature. Univariate and multivariate Cox regression analyses, time-dependent ROC analysis of 1, 3, and 5 years, and time-dependent ROC analysis of risk score and clinical features in GEO (a, c, e, g) and ICGC (b, d, f, h) cohorts.

Univariate and multivariate Cox regression analyses were applied to reveal the independent prognostic indicator value of risk score for ES. The univariate Cox regression analysis results (Figures 4(a) and 4(b)) showed that the risk score was significantly related to OS in the GEO dataset (*P* < 0.001, HR = 3.6835, and 95% CI = 2.4016–5.6496) and ICGC dataset (*p*=0.016, HR = 1.0538, and 95% CI = 1.0098–1.0997). The multivariate Cox regression analysis (Figures 4(c) and 4(d)) results showed that the risk score was an independent indicator in the GEO dataset (*P* < 0.001, HR = 4.2485, and 95% CI = 2.5937–6.9591) and ICGC dataset (*P*=0.035, HR = 1.0480, and 95% CI = 1.0032–1.0947).

Moreover, the accuracy of the signature was assessed through ROC analysis. The outcomes (Figures 4(e) and 4(f)) showed that 1-, 2-, and 3-year AUC values for the GEO cohort were 0.0856, 0.810, and 0.834, while those for ICGC cohort was 0.0833, 0750, and 0.718, respectively. The risk score values and the clinical features for OS were compared (Figures 4(g) and 4(h)), and the results indicated that the risk score was the best predictor.

All the outcomes demonstrated that the MTG signature was of good prognostic prediction power for ES overall survival.

### 3.4. The Efficiency of the MTG Signature in the GEO Cohort

**Figure 5:**
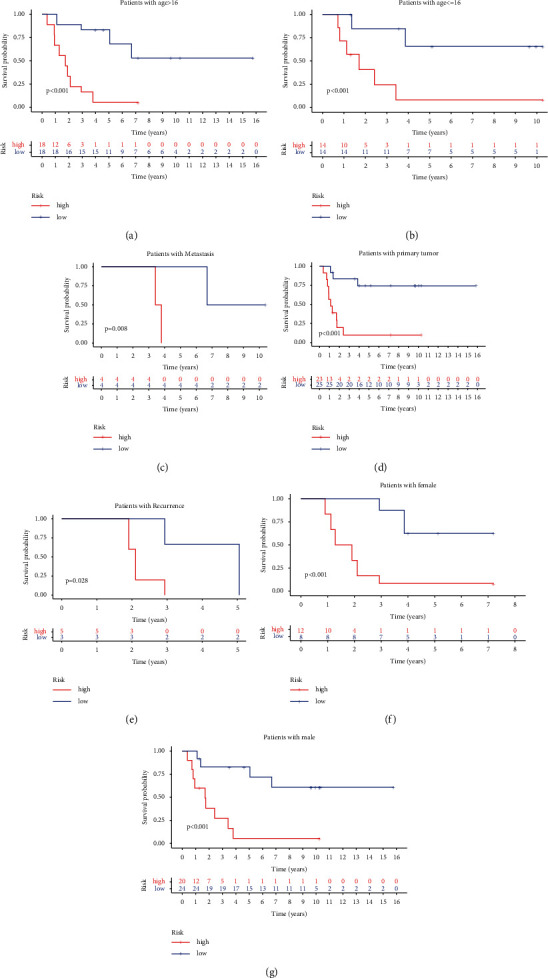
The efficiency of the MTG signature for prognosis of different subgroups in the GEO cohort. K-M survival analysis for the low- and high-risk groups categorized by clinical variables, comprising age (a, b), metastasis (c), primary tumor (d), recurrence (e), and gender (f, g).

To explore the efficiency of the signature, ES patients with different clinical features were divided into different groups based on age, gender, and disease state (primary tumor, metastasis, and recurrence). All the outcomes (Figure 5) showed that the high-risk group was significantly related to poorer OS (*P* < 0.05).

**Figure 6:**
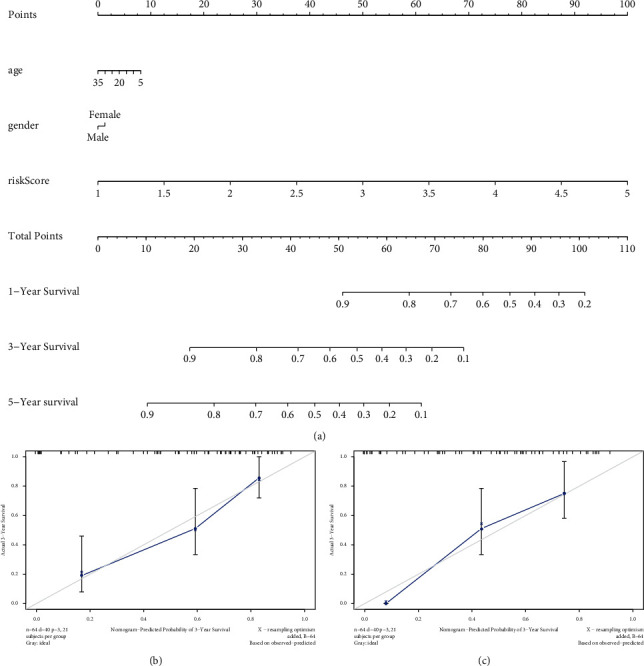
The nomogram for predicting the survival of ES patients in the GEO cohort (a) and 3-year (b) and 5-year (c) survival predicted by calibration plots.

We also applied the nomogram to explore the 3- and 5-year OS of ES patients based on the risk score and other clinical variables (Figure 6(a)). The calibration curve demonstrated satisfactory performance for 3 and 5 years in ES patients (Figures 6(b)–6(c)).

### 3.5. Evaluation of Immune Cell Infiltration and Immune Checkpoints

**Figure 7:**
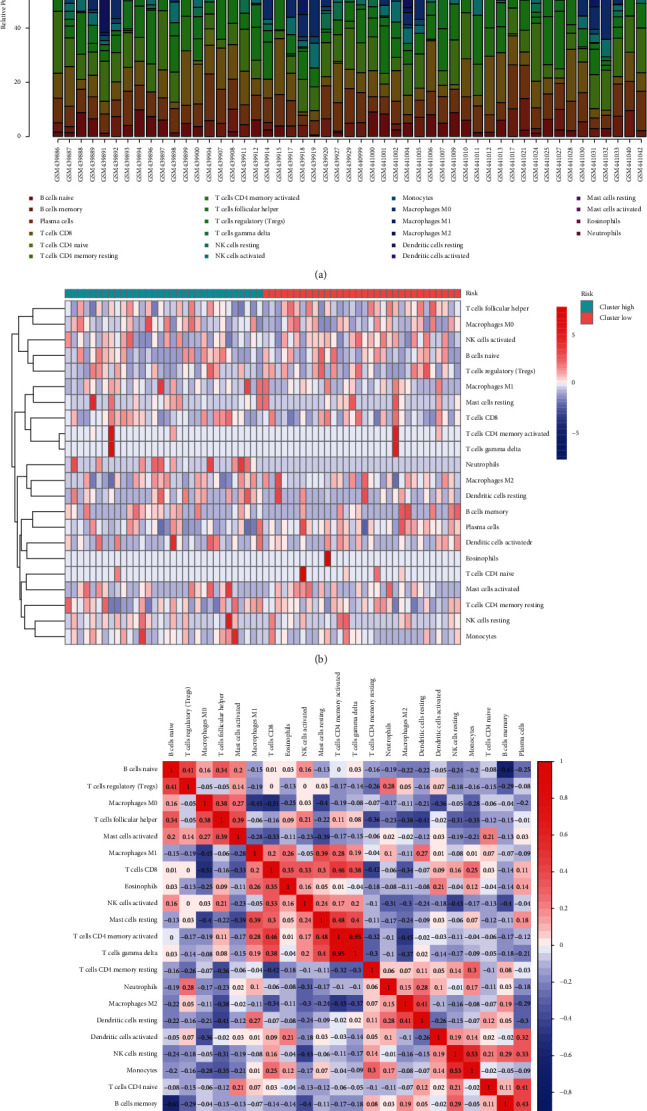
Twenty-two immune cells proportion (a), heatmap (b), and correlation (c) analysis in the GEO cohort.

The relationship between our MTGs and immune infiltration was assessed with the CIBERSORT algorithm. The infiltration profiling and the heatmap of the two groups of 22 immune cells are displayed in Figures 7(a) and 7(b). The correlation of the 22 immune cells is illustrated in the heatmap (Figure 7(c)).

**Figure 8:**
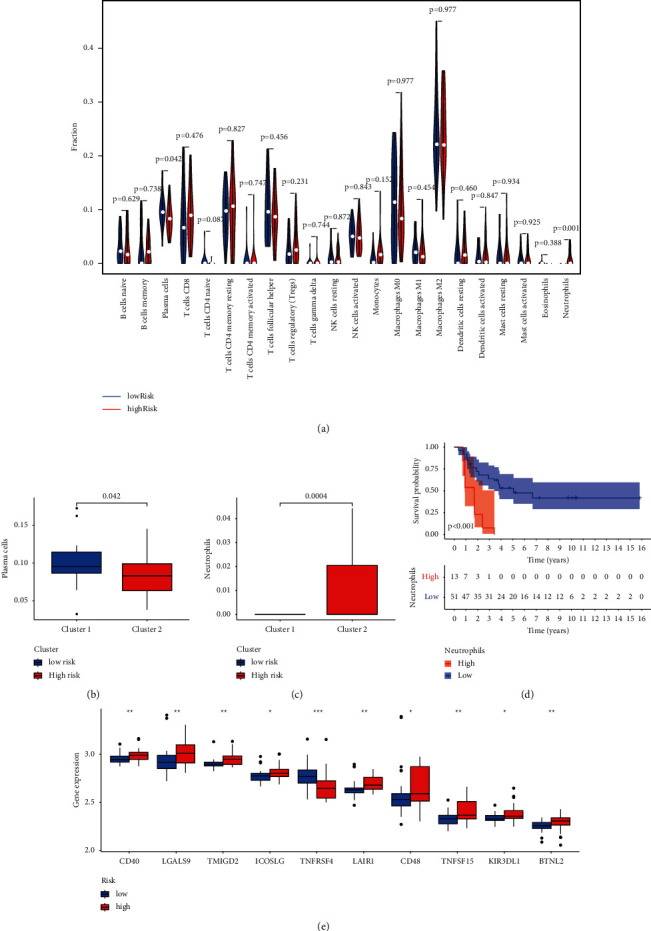
Evaluation of immune cell infiltration and immune checkpoints. (a) Violin plot showing the composition of immune cells between the low- and high-risk groups (low: blue vs. high: red). Box plot showing the differential immune cells: neutrophils (b) and plasma cells (c). (d) K-M survival analysis of the neutrophils in the low- and high-risk groups. (e) Box plot showing differential expression of immune checkpoints genes.

Neutrophils (Figures 8(a) and 8(c)) were highly expressed in the high-risk group (*P* < 0.001). Plasma cells (Figure 8(b)) were highly expressed in the low-risk group (*P*=0.042). According to the Kaplan–Meier curves (Figure 8(d)), ES patients with higher neutrophil levels exhibited a poorer survival rate.

As for the relationships between immune checkpoint genes (Figure 8(e)) and MTG signature, we found CD40, LGALS9, TMIGD2, ICOSLG, LAIR1, CD48, TNFRSF15, KIR3DL1, and BTNL2 were all significantly highly expressed in the high-risk group while TNFRSF4 was lowly expressed.

## 4. Discussion

Ewing sarcoma is one of the most aggressive sarcomas. Merely 30% of ES patients with metastasis survive [13]. The lung is one of the most common sites suffering from Ewing sarcoma metastasis while the previous study also showed that colonic Ewing sarcoma could cause liver metastasis [14]. Early diagnosis and treatment can remarkably improve the clinical prognosis of ES, which justifies the need to seek effective biomarkers for the early diagnosis and treatment of ES. Compelling evidence is in support of the finding that MTGs play crucial roles in the development and progression of ES. MTGs have immense potential as promising therapeutic targets and prognostic predictors. However, studies on the prognostic value exploration of MTGs are immature.

Moreover, the outcomes of the survival status and K-M curve demonstrated that the risk score was significantly related to a poorer survival rate in GEO and ICGC datasets. Based on univariate and multivariate Cox analyses, we found that the risk score was of great value as an independent prognostic predictor. ROC curves revealed that our signature could accurately predict the prognosis of ES patients in the two cohorts. Validation procedures showed that the efficiency of the signature was satisfactory in patients with different clinical features. The constructed nomogram could predict the 1-, 3-, and 5-year survival probabilities, which might be useful for personalized treatment. Taken together, all the outcomes indicated that the MTG signature was of good robustness for predicting the prognosis of ES patients. Ren et al. [2] had previously identified immune cell infiltration had a close correlation with ES. Here, we adopted CIBERSORT to explore the roles of the infiltrating immune cells in our signature. The results showed that neutrophils and plasma cells were differentially expressed in the high- and low-risk groups. Of note, neutrophil cells were significantly related to poorer OS, which was not the case for plasma cells.

The six genes were identified as follows: PC, CHPT1, and PYGB were oncogenes, while DGKA, CPT1A, and NUDT12 were protective genes. Studies on the relationships between the six genes and Ewing sarcoma are immature. In several cancer tissues, including mammary, lung, gallbladder, and thyroid, Kiesel et al. found that PC was overexpressed as compared with the normal tissue [15]. Other pieces of evidence indicate that PC exerts crucial effects in metastasis, particularly because it connects many metabolism pathways, whereas metastatic tumor cells show an increased need for redox defense and ATP. Mounting evidence shows that CHPT1 is a curative target for prostate cancer [16] and is related to stemness and trastuzumab resistance in breast cancer [17]. Elsewhere, PYGB was also reported to be upregulated in numerous tumors, including gastric cancer, lung cancer, ovarian cancer, and renal cell cancer [18]. Studies have also revealed a close correlation of DGKA, CPT1A, and NUDT12 with numerous tumors [19–21].

Appling a nomogram to cancer prognosis can allow for the interpretation of prediction models and the establishment of numerical possibilities for individualized treatment [22]. We integrated the risk score with other clinicopathological features and established a novel nomogram to assist clinical decision-making. Fang and Chen [23] recently established a nomogram based on the autophagy-related genes, which embodied a favorable effect in hepatocellular carcinoma. In another study, a nomogram containing clinicopathological features and the MTG signature exhibited good results for LUAD prognosis predicting [1]. These data are in support of our nomogram which demonstrated good predictivity potential for 1-, 3-, and 5-year survival of ES patients. In addition, increased studies had revealed the relationship between tumor metabolism and tumor immune [24, 25]. As one of the immune infiltration cells, neutrophils served as prognosis-related cells and were found to be overexpressed in the high-risk group. According to the immune checkpoint genes, the high-risk group was mainly positively related to the expression levels of immune checkpoint genes.

Although our study presents valid clinical significance, a few limitations cannot be ignored. To begin with, the GEO and ICGC cohorts were derived from the public database, and the clinicopathological features in the two cohorts were incomplete and limited. As such, we needed our dataset to show the effectiveness of the MTG signature. Besides, we did not identify the detailed molecular mechanisms of each MTGs in ES, and further studies are warranted to analyze the details. Lastly, the detailed relationships between the risk score and immune infiltration should be addressed in future.

## 5. Conclusion

In conclusion, the MTG signature developed in this work displayed an upstanding performance as an independent factor for predicting the prognosis of ES patients. The signature has been validated in an independent cohort. Also, the MTG signature-related nomogram can predict 3- and 5-year survival outcomes accurately. Overall, this study presents six genes with potential roles as prognostic indicators and therapeutic targets for ES.

## Figures and Tables

**Table 1:** The characteristics of ES patients in the two datasets.

Variables	GEO cohort	ICGC cohort
Numbers	64	55

Age, years (%)		
>16	36 (56.25%)	26 (47.27%)
≤16	28 (43.75%)	29 (52.73%)

Gender (%)		
Female	20 (31.25%)	24 (43.64%)
Male	44 (68.25%)	31 (56.36%)

Survival status (%)		
Alive	24 (37.5%)	27 (49.09%)
Dead	40 (62.5%)	28 (50.91%)

## Data Availability

The datasets analyzed in this study can be derived from https://www.ncbi.nlm.nih.gov/geo/query/acc.cgi?acc=GSE17679 and https://dcc.icgc.org/repositories. The codes in this study are available from the corresponding author on reasonable request.

## Abbreviations

ES: Ewing sarcoma
LDH: Lactate dehydrogenase
DEGs: Differentially expressed genes
MTG: Metabolism-related gene
ICGC: International Cancer Genome Consortium
ssGSEA: Single-sample gene set enrichment analysis
LASSO: Least absolute shrinkage and selection operator
GSEA: Gene Set Enrichment Analysis
OS: Overall survival
PCA: Principal component analysis.

## Supplementary Materials

Supplementary Materials 944 metabolism-associated genes are shown.
